# Elementary Principles of Electro-Therapeutics for the Use of Students

**Published:** 1884-08

**Authors:** 


### Book Reviews.

ELEMENTARY PRINCIPLES OF ELECTRO-THERAPEUTICS, FOR THE USE OF PHYSICIANS AND STUDENTS. With 135 illustrations, prepared by C. M. Haynes, M. D., published by the McIntosh Galvanic and Faradic Battery Company, Chicago, Ill.

The title of this new candidate for public favor is calculated to inspire an interest in the work, as the advances made in these latter years by those engaged in the application of electricity to the cure of various disorders, create a demand for the kind of information that ought to be contained in what claims to be a plain, practical and complete treatise on Magnetism, Franklinism, Galvanism and Faradism.

It devolves upon us to state at the outset that while the student or practitioner must be presumed to have some knowledge of the cardinal doctrines and facts connected with electrical phenomena, it is desirable to have a clear and concise description of the instruments used, and the principle upon which they are applied in the treatment of diseases, with a general outline of the affections to which electro-therapeutics may be appropriate. Let us proceed to see in how far this work meets the requirements of such a handbook for students and practitioners:

The introduction, like all productions of its class, contains generalizations and bibliographical notes,which do not facilitate a better understanding of the subject. The reader does not profit by the torpedo cure of the Greek slave of Anthero, nor derive benefit from the virtues of the amber beads of the Grecian women. The kite experiment of Franklin, as that of Dufaye, with a cord of moist packthread, with the rudimentary telegraph of LeSage, are among the elementary experiences which do not require recapitulation in a practical illustration of the fruits of our progress in electro-therapeutics. There is no sort of foundation for the regret of the author that the early observers left us no fuller accounts of their experience with disease, as their reports of marvelous cures with electricity do not indicate any important result in a scientific point of view. It is no less a matter of surprise that reference is made in such a work to the electro-galvanic theory of yellow fever, as all who take the trouble to read it will concur in the verdict that it lacks every element of sound medical philosophy based upon scientific principles.

The allusion to a grave suggestion that the hairy coat of animals and the dense oily skin of the negro, are preservatives from malaria, serves no other end that is apparent, than to place the author on the same platform with the lamented Turnipseed, in claiming that the hymen in the negro race is not at the entrance of the vagina, and insists that this is "one of the anatomical relations Providence has given us to show the non-unity of the races."

There is one redeeming clause, however, in the introduction, inculcating that many reports of cases as now made are worthless in not being sufficiently explicit. "In reporting cases these points should be distinctly brought out, after describing the disease in the usual manner:

1. The method of applying the current, whether general or local, labile or stabile, continuous or interrupted.
2. The kind of current used.
3. The direction of the current and location of the electrodes.
4. The length of sitting.
5. The number of sittings.
6. The interval between sittings.
7. The power of battery current employed."

Independent of the omission of all records in the use of static electricity, this schedule lacks the very essential element of clinical observation in giving no detail of the effects. It would not be amiss in another edition to fill out this enumeration, and eliminate all else from the introduction, retaining the vocabulary.

In passing to the body of the book there are sundry details connected with magnetism, of which the first chapter treats, and among the authors quoted in regard to the influence of terrestial magnetism upon the nervous system, it is an unpardonable omission that no allusion is made to the work of Reichenbach, entitled electro-dynamics or odic force. This elaborate and painstaking investigation throws more light upon the relations of electricity to the human body than any of the more recent undertakings in this department, and if the book should be out of print no more acceptable service could be rendered to those interested in neuro-pathology as influenced by terrestial electricity, than its republication at an early day.

As the normal capacity of the human body for developing electricity should be taken into the account of the influence of different appliances, the author has done an acceptable service in the second chapter under the head of animal electricity, to present the conclusions from Hemmer's experiments:

1. "The human body always possesses electricity, but its strength is not the same in all, in some it is positive and in some negative.

2. The intensity and nature often varies in one and the same person.

3. The natural electricity of the body is positive, for this is always its character when there has been no violent exertion.

4. This normal positive electricity is changed into negative by exposure to cold, or else is greatly enfeebled.

5. The same change occurs from over exertion or lassitude.

6. The natural electricity is also changed into negative by sudden, rapid and violent motion.

7. Prolonged mental exertion increases positive electricity.

8. Positive electricity is increased in winter and diminished in summer, ceasing entirely during perspiration.

9. This electricity is not due to the friction of the clothing, since it was still observed after remaining for hours on an insulating stool."

A very striking verification of the charging of the body with electricity is the parlor experiment of a lady passing briskly over the carpeted floor and extending a finger to a gas burner, so as to pass a spark to the metal tube or beak and thus ignite the gas.

After some bibliographical history, which, if not quite irrelevant is certainly unnecessary, a very intelligible and appropriate description of the mode of applying Franklinic electricity is presented in the latter part of the second chapter, which is closed with the following judicious precepts:

1. "Give electricity at first in its mildest form, and increase it gradually as the patient can bear.

2. Use electrodes attached to long insulating handles.

3. Do not neglect the employment of other means while electricity is being tried."

The third chapter, which is on galvanism, has most of the recognized practical demonstrations for the different modes of developing this form of electricity, and but for the rather obscure disquisition upon electric measurement and the laws for determining the strength of currents, affords a satisfactory presentation of the adaptation of the direct continuous current to diseases. The recent application of this process in extra-uterine pregnancy has escaped the notice of the author and promises most satisfactory results in the early stage, to arrest the development.

As an explanation of the humbuggery practiced by the vendors of electrical apparatus, the author states that "minute quantities of electricity are sufficient to disturb the position of a compass-needle; and this fact is frequently taken advantage of by dealers in various so-called electrical appliances to convince the ignorant of the enormous amount of electricity furnished by them. A magnetized pen-knife will disturb the needle equally as much, and possesses fully as much therapeutical power as many of the electrical devices put on the market in the guise of belts, jackets, discs, etc., etc."

The metallic attachment to the McIntosh uterine supporter cups for the purpose of conveying a galvanic current directly to the cervix uteri is well delineated in a wood cut, and yet the original abdominal spring pessary devised by the author of this review, and presented with drawings to the South Carolina Medical Association, which appeared with their transactions published about 1854, afforded a more convenient form of applying electricity to the vagina and uterus, as the concave tubular support of the womb was of silver with a steel spring that attached it to the front pad of the abdominal supporter. The form of that pessary was subsequently used by various manufacturers of hard rubber goods, as shown in the McIntosh cup, and if any one should be interested to verify the details as to the first instrument, I would state that it was made by Mr. C. Heinz, then of Columbia, S. C., and now in business at No. 15 Whitehall street, Atlanta, Ga.

The claim of priority for this mode of constructing and applying a pessary has not been preferred at an earlier period, as no occasion demanded it.

The fourth chapter treats of electrolysis, whose laws are expressed by the amount of decomposition that takes place in any given case owing to the strength of the current, the nature of the substance acted upon, and the material of which the electrodes are composed. It has proved most effective in erectile and small cystic tumors, and relief has been afforded in cases of urethral stricture.

Galvano-cautery is the subject of the fifth chapter, and in a few pages gives the advantages and disadvantages of this process, which has a special adaptation to a certain class of cases only. It is doubtful whether all may not be done by the thermo-cautery quite as efficiently and with less trouble, except in case of ligatures.

Faradism occupies the sixth chapter, and the induction currents are employed according to Duchenne, by four methods :

1. Faradization of the skin.
2. Direct muscular faradization.
3. Indirect muscular faradization.
4. Faradization of the internal organs and special senses.

A knowledge of the motor points, as demonstrated by Ziemssen, is now regarded as essential to the scientific faradization of the muscles.

The seventh chapter is a special recommendation of the McIntosh Combined Galvanic and Faradic Batteries, around which it is fair to conclude all the interest of this work is polarized by its duple electric energy.

But if this house understands the art of bringing their wares into the market by legitimate representation of their qualities, and they prove to meet a want satisfactorily, there can be no valid objection to giving the establishment the benefit of its intelligent enterprise.

Those who wade through the electro-thermal baths of the eighth chapter will be still further impressed with the skill of the author in advertising for his employers, and be prepared to enter in earnest upon the study of electro-physiology, electro-diagnosis and electro-therapeutics, which fill up the rest of the volume.

The application of electricity in certain conditions of the nervous system connected with impotency do not receive the attention their importance warrants, owing perhaps to the small display of apparatus that is requisite.

The hand book of electro-therapeutics, by Dr. William Erb, has been translated and issued as a part of Wood's Library for 1883, and with thirty-nine wood cuts and 366 octavo pages,gives similar information to that which our author furnishes, and hence the door is not closed to those in search of a guide in the prosecution of electric researches.

Without any just claim to consideration as a scientific text-book, this volume of Dr. Haynes contains much that is useful and will prove a good companion in the use of instruments.

The only practical question for solution is one of finance as to information outside of the notice of these instruments, being a fair and just equivalent for the $2.00 charged for this book of 426 pages well printed and on good paper. J. McF. G.

